# Precision medicine in breast cancer: reality or utopia?

**DOI:** 10.1186/s12967-017-1239-z

**Published:** 2017-06-17

**Authors:** Ali Bettaieb, Catherine Paul, Stéphanie Plenchette, Jingxuan Shan, Lotfi Chouchane, François Ghiringhelli

**Affiliations:** 1 Laboratoire d’Immunologie et Immunothérapie des Cancers, EPHE, PSL Research University, 75000 Paris, France; 2 LIIC, EA7269, Université de Bourgogne Franche Comté, 21000 Dijon, France; 3 Laboratory of Genetic Medicine and Immunology, Weill Cornell Medicine-Qatar, Education City, Qatar Foundation, Doha, Qatar; 4 Département d’Oncologie Médicale, Centre Georges-François-Leclerc, 21000 Dijon, France; 5 Plateforme de Transfert en Biologie Cancérologique, Centre Georges-François-Leclerc, 21000 Dijon, France; 6 UMR 1231 Inserm-Université de Bourgogne Franche Comté, UFR des Sciences de Santé, 21000 Dijon, France; 7 Université de Bourgogne, 21000 Dijon, France; 8 Immunology and Immunotherapy of Cancer Laboratory, EA7269, Université de Bourgogne, EPHE 7 Bd Jeanne d’Arc, 21079 Dijon, France

**Keywords:** Breast cancer, Molecular subtypes, Triple-negative, HER2+, Tumor heterogeneity, Precision medicine, Chemotherapy, Immunotherapy, Phase III clinical trials

## Abstract

Many cancers, including breast cancer, have demonstrated prognosis and support advantages thanks to the discovery of targeted therapies. The advent of these new approaches marked the rise of precision medicine, which leads to improve the diagnosis, prognosis and treatment of cancer. Precision medicine takes into account the molecular and biological specificities of the patient and their tumors that will influence the treatment determined by physicians. This new era of medicine is accessible through molecular genetics platforms, the development of high-speed sequencers and means of analysis of these data. Despite the spectacular results in the treatment of cancers including breast cancer, described in this review, not all patients however can benefit from this new strategy. This seems to be related to the many genetic mutations, which may be different from one patient to another or within the same patient. It comes to give new impetus to the research—both from a technological and biological point of view—to make the hope of precision medicine accessible to all.

## Background

Breast cancer, the most common cancer among women worldwide, is a highly complex, heterogeneous and multifactorial disease. A number of recognized risk factors contribute to develop breast cancer, including hormone reproduction, age, obesity, alcohol, radiation, benign breast disease and lack of exercise [1]. Above all, genetic factors play an important role in both sporadic and familial breast cancer. A meta-analysis of 52 studies revealed that breast cancer incidence is twice as much in women with a first-degree relative [2]. The similar results were also showed by twin studies [3]. Therefore, identification of breast cancer (BC) susceptibility genes has significantly improved practice for care of patients suffering cancer. A major example is the emergence of precision medicine that relies on the development of new strategies targeting altered genes involved in carcinogenesis and treatment inefficacy.

Precision medicine includes two different approaches, namely, stratified medicine and personalized medicine. The first consists of testing a drug in a cohort of patients defined by a specific molecular alteration, while the personalized medicine determines whether the concept of individualized treatment improves outcomes in all population [4].

This practice has emerged following treatment failures that are related in particular to the tumor heterogeneity and the resurgence of gene mutations not targeted by the applied therapies.

## Breast tumor heterogeneity

Decades of research have shown that tumors including breast cancer are heterogeneous. Two main types of heterogeneity are described: (i) inter-tumor heterogeneity e.i. cancers from the same primary site of origin which have distinct clinically relevant biologic differences (different histologic or molecular subtypes) that results in very heterogeneous behavior within one cancer type (ii) spatial and temporal intra-tumor heterogeneity reflecting increase of variability in gene mutations within a single tumor or during tumor growth and progression. This variability can also be observed between the primitive tumor and its metastasis. In addition, chemotherapies, by their capacity to induce DNA breaks and genetic mutations, could also enhance the number of mutations and thus increase intra-tumor heterogeneity [5]. Consequently, tumor heterogeneity increases the strength of tumors and makes treatment success more difficult to reach [6]. As discussed below, despite the development of new anti-cancer therapies targeting specific molecular defects found in BC cells, high variability response and modest clinical benefit are observed [7]. Therapy failure may be related to targeted genes that are more expressed on tumor cells and not those at low frequency in the primary tumor or occurred during treatment [8].

It is well known that the characteristics of BC differ among various patients [9]. Differences in clinical presentation are observed such as size, scalability and multifocality. There are also many histological differences with variability in type, grade, mitotic index and Ki67. According to the WHO 2012, for the breast, many histotype are defined and reported [10]. This heterogeneity is not only observed among patients but also in metastases compared to the primary tumor. Indeed, substantial genomic changes often occur in disease progression from primary to metastasis. Shah et al. reported a whole genome sequencing of an advanced invasive breast cancer case, which demonstrated the existence of 19 non-synonymous mutations present in the metastases but not in the primary tumor, illustrating the spatial and temporal dynamics of intratumoral heterogeneity [11]. In another study, these discordances also observed between the primary carcinoma and metastases [12], could explain why biomarkers measured exclusively from the primary tumor may not be informative enough for predicting responsiveness to therapy. This discordance in gene or protein expression between primary and recurrent BC is link to estrogen receptor (ER), progesterone receptor (PgR) and the oncogenic human epidermal growth factor receptor 2 (HER2) and may include other potential drug targets. There is also a high level of discordance in phosphatase and tensin homolog (PTEN) level, and phosphoinositide-3-kinase catalytic alpha polypeptide (PIK3CA) mutations between primary tumors and metastases that may influence patient selection for targeted therapies [13].

Thus, it is the genomic analysis and characterization of several biomarkers that has revolutionized the clinical management of patients. As we will develop below, these gene alterations represent novel target to engineer new therapies for BC.

## Molecular subtypes of BC and precision medicine

Thanks to many new techniques of analysis, using microarray or the more recent genomic revolution [14], several thousands of genes and their expression can be studied. This allowed re-classifying the tumors into different groups (Table 1):

**Table 1:** Rate and specificities of the breast cancer molecular subtypes

Subtypes	Frequency	Gene expression	Histologic grade level	Prognosis
Luminal	60% BC			
Luminal A		ER^+^, PR^+^, HER^−^	Low	Good
Luminal B		Low p53 mutation ER^+^, PR^+^, HER2++	High	Less favorable
Basal-like (TN)	15–20%	ER^−^, PR^−^, HER^−^	Often grade III	Poor (chemosensitive)
HER2^+^	25%	HER2++	High	More aggressive (anti-HER2 sensitive)

*TN* triple negative, *BC* breast cancer, *ER* estrogen receptor, *PR* progesterone receptor, *HER2* human epidermal receptor 2, *HER++* overexpression of HER

- ER-positive (ER+) group which was subdivided into two distinct prognostic groups, luminal A, HER2-negative (HER2−), Ki67 protein low, and PgR high, and luminal B, HER2−, and either Ki-67 protein high or PgR low [15].
- Triple-negative BC: ER-negative (ER−), PgR-negative (PgR−), and HER2− tumors [16].
- HER2-positive (HER2+) BC exhibiting amplified HER2/neu. This cancer is particularly aggressive [17].
- A more recently described class, claudin low, often triple-negative, but distinct in that is low expression of cell–cell junction proteins including E-cadherin [18].

## The benefit of molecular subtyping in the case of surgery, radiotherapy or chemotherapy for breast cancer

To evaluate the impact of the surgical treatment and outcome according to the molecular subsets of BC, Mazouni et al. analyzed 1194 patients treated for primary BC. They showed that molecular subsets exert an impact on breast-conserving surgery (BCS) and nodal surgery rates. BCS is more favorable in luminal A (70.6%) than triple-negative (66.2%) and HER2+ tumors (60%). Nodal positivity was more frequent in HER2 and luminal B subtypes (p < 0.001) [19].

Other groups have evaluated the impact of molecular subtypes on local–regional control in different patient populations. In a previous report, Nguyen et al. analyzed 793 patients with BC treated with breast-conserving therapy (BCT) consisting of lumpectomy and radiation therapy. They showed that local recurrence was particularly low for the luminal A subtype (hormone receptor (HR)+/HER2−), but was less than 10% at 5 years for all subtypes [20].

Molecular subsets could also serve to identify patients at high risk of local–regional recurrence (LRR) in patients with BC that received neoadjuvant chemotherapy and then underwent BCT. In a cohort of 595 breast cancer patients, Caudle et al. showed that patients with HR+/HER2− and HR+/HER2+ subsets had excellent LRR-free survival regardless of tumor response to neoadjuvant chemotherapy. In contrast, patients with HR−/HER2+ and HR−/HER2− subsets with poor response to neoadjuvant chemotherapy had worse LRR-free survival after BCT [21]. This is consistent with previous published data from the multicenter I-SPY 1 TRIAL evaluating patients (221) with ≥3 cm tumors by using early imaging and molecular signatures, outcomes of pathologic complete response (pCR) and recurrence free survival. In this study the pCR rates were 9 and 35% in patients with HR+ or HR− tumors, respectively [22]. Guarneri et al. also reported that patients with ER− tumors were more likely to achieve a pCR after neoadjuvant chemotherapy [23].

According to the St Gallen 2015 recommendations [24], the decision of systemic adjuvant therapies for primary BC should be based on the surrogate intrinsic phenotype determined by ER/PgR, HER2 and Ki67 assessment. All luminal cancers should be treated by endocrine therapy. The majority of luminal A tumors, except those with high risk of relapse, require no chemotherapy, while luminal B HER− tumors need endocrine therapy and chemotherapy for the majority of cases (Table 1).

Endocrine therapy is also recommended for ER+ metastatic breast cancer (MBC) including drugs that suppress estrogen production as aromatase inhibitors (AI) and direct inhibitors of estrogen receptor [25]. Indeed, AI (e.g. letrozole and anastrozole) have become the treatment of choice in first-line therapy in postmenopausal patients suffering from BC that is HR+ and HER2− [26]. Unfortunately, more than one-third of patients do not benefit from endocrine therapy due to intrinsic resistance. Furthermore, even though the antitumor benefit of these drugs, resistance often emerges after prolonged exposure [27–29]. Several mechanisms have been proposed to explain this resistance such as activation of the Pi3K/Akt/mTOR pathway or mutations in the gene encoding ERα, estrogen receptor gene (ESR1) (Y537S, E380Q and D538G mutations) that render estrogen receptor constitutively active and confer partial resistance to currently available endocrine treatments of patients with metastatic lesions [30–33]. In a phase III BOLERO-2 study, everolimus an inhibitor of Pi3K-mTOR pathway, was used with exemestane, an AI inhibitor, in patients who progressed on AI therapy. This combination significantly improved disease-free survival (DFS) compared to exemestane alone [34]. Since everolimus can activate mTORC2 and Akt [35], some clinical trials using Pi3K or Akt inhibitors have been designed in endocrine-resistant advanced BC. In this context, adding a PI3K inhibitor to anti-ER therapy may be an attractive treatment option for women with advanced HR+ BC that becomes resistant to endocrine therapy, according to findings from the phase III BELLE-2 trial [36]. In a secondary analysis of the BOLERO-2 study, a large study providing overall survival data with respect to ESR1 mutations has been conducted [37]. In this study, of 541 evaluable patients, 156/541 (29%) had ESR1 mutation D538G (21.1%) and/or Y537S (13.3%), and 30 had double-mutations, indicating that ESR1 mutations are prevalent in ER+ AI-treated metastatic breast cancer. Another study conducted in a relative large cohort of MBC patients, using droplet polymerase chain reaction (dPCR) on circulating tumor DNA (ctDNA), showed that ESR1 mutations in patients, with MBC receiving prior AI treatment, was observed in 18/128 cases (14%), with D538G mutations but 21% of patients carry ESR1 polyclonal mutations. In contrast, ESR1 mutations were not observed in patients who had only received tamoxifen treatment [38], further demonstrating that acquisition of ESR1 mutations is more common after treatment with AI. Patients with ESR1 mutations had a substantially shorter progression-free survival on subsequent AI-based therapy [38]. However, ESR1 mutations are not exclusively found following AI treatment. Wang et al. reported that, applying digital droplet PCR (ddPCR), ESR1 mutations were found in 3/43 primary tumors, 1/12 bone metastatic lesions and 3/38 brain metastasis tissues in ER− positive MBC patients [39].

Dysregulation of the cell cycle control involving components of CDK4/6 and cyclin D, which is a frequent event in BC, represents another mechanism that may cause resistance to anti-hormonal treatment of BC. Thus, selective CDK4/6 inhibitors represent a significant therapeutic advance in HR+ BC. In a randomized phase 2 clinical trial PALOMA-1/TRIO-18, Finn et al. have assessed the safety and efficacy of the CDK4/6 inhibitor palbociclib in combination with letrozole as first-line treatment of patients (n = 165) with advanced ER+, HER2− BC. They showed that the addition of palbociclib to letrozole significantly improved progression-free survival in patients. The common grade 3 or 4 adverse events were neutropenia and leukopenia [40]. This was followed by a phase III clinical trial PALOMA-2 including 666 patients with previously untreated ER+, HER− advanced BC. They confirm that palbociclib combined with letrozole resulted in significantly longer progression-free survival (24.8 months) than with letrazole alone (14.5 months) [41]. In addition, the analysis of ER expression, cyclin D1 (CCND1), retinoblastoma (RB) and P16 (CDKN2A) status, as well as Ki67 expression, did not result in the identification of sub-populations who did not benefit from the addition of palbociclib to letrozole. By H-score (hazard ratio for disease progression or death), some variation between subgroups was observed, but the addition of palbociclib to letrozole improved progression-free survival (PFS) across all sub-populations. Although the most common grade 3 or 4 adverse events were neutropenia (occurring in 66.4% of the patients), and leukopenia (24.8%) in the palbociclib-letrozole group. The findings of this study are consistent with another phase III clinical trial PALOMA3, in which Turner et al. also assessed the efficacy of palbociclib and fulvestrant, an anti-estrogen, in advanced HR+/HER− BC (involved 551 patients). The authors showed that the PFS median was 9.2 months with palbociclib–fulvestrant and 3.8 months with placebo–fulvestrant. Similarly to Finn’s results, the most common grade 3 or 4 adverse events in the palbociclib–fulvestrant were neutropenia (62%), and leukopenia (25.2%) [42]. So far no data are available yet for overall survival (OS) with palbociclib. Although the results with palbociclib show the improvement in PFS, neutropenia-associated palbociclib therapy has an impact on life quality, even if it is managed appropriately. Turner’s group also reported that combination of palbociclib and fulvestrant appeared to be equally effective in patients with or without ESR1 mutations analyzed in plasma from the PALOMA trial [43]. Further studies are required to confirm the efficacy of CDK4/6 inhibitors in ESR1 mutant cancer. Recently, Hortobagyi and colleagues [44] assessed the efficacy and safety of the selective CDK4/6 inhibitor ribociclib combined with letrozole for first-line treatment in 668 postmenopausal women with HR+, HER2− recurrent or MBC who had not received previous systemic therapy for advanced disease. They found that the duration of progression-free survival was longer in the ribociclib group than in the placebo group. Even more, the overall survival response rate was 52.7% in ribociclib group vs. 37.1 for the placebo. As for palbociclib, common grade 3 or 4 adverse events that were reported in 10% of the patients were neutropenia, 59.3% in the ribociclib group vs. 0.9% in the placebo group and leukopenia (21.0% vs. 0.6%).

Chemotherapy and anti-HER2 treatments are recommended for HER2+ patients. As for the triple-negative BC (TNBC), it could benefit from adjuvant chemotherapy.

## HER2+ advanced BC

About 20% of invasive BC overexpress HER2. Gene amplification of this receptor is associated with increased metastatic potential and decreased overall survival (OS) with further heterogeneity during the evolution of MBC [45, 46]. The follow-up of the HER2 expression during the course of the disease is made possible thanks to the circulating tumor cells (CTC) and liquid biopsy estimation [47]. The presence of CTC has been associated with poor prognosis in patients with early stage of BC [48], and those with metastases [49, 50]. In addition, HER2+ CTC is associated with worse DFS and OS [51]. These and other studies have led to the development of treatment of patients with HER2+ breast tumors.

Targeted HER2+ MBC therapy was developed mainly using trastuzumab, a humanized monoclonal antibody directed against HER2-extracellular domain and lapatinib, an intracellular tyrosine kinase inhibitor that blocks HER2 and EGFR (epidermal growth factor receptor) activation [52]. Other treatments have also been recommended such as pertuzumab, a HER2 inhibitor, and taxanes as first-line treatment and trastuzumab emtansine drug conjugate as the preferred second-line treatment for advanced HER2+ BC [53]. However, despite the benefit provided by this therapy taken alone or in combination with chemotherapy, unfortunately over one/third of patients develop resistance. The occurrence of therapeutic resistance could be explained by several mechanisms. For instance, reactivation of HER family signaling pathway or the activation of alternative survival pathways such as insulin-like growth factor-1 receptor (IGF-1R) [54], and the proto-oncogene MET [55], which have been showed to dimerize with HER receptors to activate the phosphorylation cascade, appear as a potential mechanism of resistance to trastuzumab [56]. HER3, a member of EGFR family, could also dimerize with HER2 and plays a vital role in the tumorigenesis, drug-resistance and tumor progression of HER2+ BC. The upregulation of HER3 activity provides an alternate “escape route” via which tumor cells bypass either the inhibition of the HER family receptors or the inhibition of the downstream PI3K-AKT-mTOR signaling pathway [57]. This led to the CLEOPATRA phase III study, in which the combination of pertuzumab, a HER dimerization inhibitor, with docetaxel and trastuzumab regimen in MBC led to an increase of progression-free survival [58]. A series of HER2 somatic mutations are resistant to lapatinib, but are sensitive to the irreversible HER2 inhibitor neratinib [59], suggesting an alternative to overcome HER2 resistance. Among these mutations, the HER2V777L mutation seems responsible for, and a predictive marker of trastuzumab resistance [60]. In another study, a patient with BC carrying the HER2T798I mutation exhibited a sustained partial response when treated with neratinib, while HERL869R mutation is a neratinib-sensitive. In contrast, afatinib, a HER2 inhibitor, a tyrosine kinase inhibitor, can suppress HER2L869R/T798I-induced signaling and cell growth [61].

In addition to ER that can activate HER signaling [62], alterations of INPP4-B (inositol polyphosphate 3-kinase-phosphatase, type II) and PTEN (a natural inhibitor of Pi3K), or activating mutations in PIK3CA also activate HER2 and may account for resistance to therapies targeting HER2 [The cancer genome atlas network, 2012; 63]. It is for this reason that it was recommended the association of endocrine therapy and HER2 agents to treat ER/HER2-positive patients [53]. New approaches have also been developed to treat resistance to anti-HER2 therapy. These include delivery of toxins (microtubule-inhibitory agent DM1) as a conjugate with anti-HER2 trastuzumab, named trastuzumab emtansine (T-DM1). In EMILIA clinical trial including 991 patients with HER+ advanced BC, Verma et al. reported that T-DM1 significantly prolonged progression-free and overall survival with less toxicity than lapatinib (tyrosine kinase inhibitor) plus capecitabine (an anti-cancer agent that belongs to fluoropyrimidine family) in patients with HER2+ advanced BC previously treated with trastuzumab and taxanes [64]. In a phase III clinical trial, an irreversible tyrosine-kinase inhibitor of HER1, HER2, and HER4, neratinib has been tested for its efficacy and safety after trastuzumab-based adjuvant therapy in a cohort of 2840 patients with early stage HER2+ BC [65]. In this study, neratinib significantly improved 2-years invasive disease-free survival when given after chemotherapy and trastuzumab. However, no data on long-term follow-up whether this improvement in BC outcome is maintained. In order to overcome trastuzumab resistance, a phase III clinical trial has been conducted with a broader inhibition of ErbB receptors with afatinib in combination with vinorelbine in patients with HER2+ MBC that had progressed on previous trastuzumab-based therapy [66]. The authors showed that afatinib plus vinorelbine did not improve progression-free survival compared with trastuzumab plus vinorelbine in these patients. Furthermore, afatinib plus vinorelbine was less tolerable than trastuzumab plus vinorelbine.

## Vaccines

An alternative resistance pathway consists on the up-regulation of the vascular endothelial growth factor (VEGF) pathway in HER2-expressed MBC [67]. This was supported by the results of the BEVERLY2, an open-label, single-arm phase II study combining bevacizumab, trastuzumab and chemotherapy in patients with primary HER2+ inflammatory BC, which confirmed the safety and efficacy of the regimen [68].

At the same time, vaccines targeting different tumor antigens are developed and have shown an ability to induce an anti-tumor immune response. HER2 represents a promising tumor antigen for vaccination in BC.

The HER2 protein is a promising tumor antigen for vaccination in BC. The peptide E75 (Nelipepimut-S, NeuVax™) is derived from the HER2 protein. A Phase I/II trial evaluated this vaccine associated in adjuvant with the granulocyte–macrophage colony–stimulating factor (GM-CSF) in HLA-A2/3+ patients with high-grade cancer. The control group was of HLA-A2/3− patients not receiving the vaccine. A total of 108 patients, and one-third of HER2+ received the vaccine. The addition of vaccine as adjuvant in addition to standard treatment increases the DFS at five years to 89.7% vs. 80.2% in the control group with no significant side effect [69]. An adjuvant phase III trial is currently underway the patients HER2+ (NCT01479244), and a phase II evaluating the vaccine associated with trastuzumab is also in adjuvant in patients with lymph node involvement (NCT01570036).

More recently, the results of a randomized phase II trial, evaluating the peptide AE37, an epitope of HER2, in patients with BC in adjuvant situation. The trial enrolled 298 patients; 153 received AE37+GM-CSF and 145 received GM-CSF alone. No benefit in terms of DFS has been shown. However, in this study, an encouraging trend in a subgroup of 25 patients with TNBC, with a five-year DFS estimated at 77.7% in the vaccinated group versus 49% in the control group (n = 25) [70]. Further evaluation in a randomized trial enrolling TNBC patients is warranted.

## Triple-negative breast cancer (TNBC)

TNBC is a very heterogeneous disease compared to other BC subtypes, and patients with TNBC have a higher risk of disease relapse [71]. Thanks to genome-wide approaches, seven TNBC subtypes are defined, including basal-like (BL1 and BL2), an immunomodulatory, a mesenchymal, mesenchymal stem-like 1, luminal androgen receptor (LAR) and unstable [72, 73].

This heterogeneity makes treatment of this subtype of BC very difficult and ineffective. Indeed, unlike ER+ or HER2+ BC, TNBC do not benefit from endocrine therapy or trastuzumab. Chemotherapy remains the systemic medical treatment, which improves the outcome to a greater extent in this subtype of BC than in ER+ BC. Indeed, in a multicentric retrospective study, Colleoni et al. have shown that a significantly greater benefit for chemotherapy (three or six courses of adjuvant classical cyclophosphamide, methotrexate, and fluorouracil (CMF) with or without endocrine therapy versus endocrine therapy alone) was observed in TNBC (HR, 0.46; 95% CI 0.29–0.73; interaction p = 0.009 v endocrine receptor-present disease). The magnitude of the chemotherapy effect was lower in HER2+/ER− disease (HR, 0.58; 95% CI 0.29–1.17; interaction p = 0.24 vs. ER+ disease) [74]. To the extent that overexpression of EGFR is more common in TNBC, cetuximab, targeted against EGFR, has been assessed in combination with carboplatin [75]. In this randomized phase II clinical trial including 102 patients with metastatic TNBC, combination cetuximab plus carboplatin produced responses in fewer than 20% of patients, suggesting that most patients had alternate mechanisms involved in response failure to anti-EGFR.

The most interesting clinical target in TNBC is the poly(adenosine diphosphate-ribose) polymerase (PARP), an enzyme involved in base-excision repair after DNA damage. Several clinical trials have been conducted to evaluate the safety and efficacy of different PARP inhibitors in patients with MBC. In a phase II clinical trials, Tutt et al. evaluated the PARP inhibitor olaparib in a cohort of patients with MBC with BRCA1 et 2 mutations. In TNBC, the response rate was 54% (7/13) in patients treated with 400 mg olaparib, and 25% (4/16) in patients treated with 100 mg olaparib [76]. In a phase III clinical study including 519 patients with recurrent TNBC treated with iniparib, a PARP inhibitor, in combination with gemcitabine and carboplatin regimen, O’Shaughnessy et al. showed that in the primary analysis, no statistically significant difference was observed for OS. An exploratory analysis showed that patients in the second-/third-line had improved OS [77]. In a phase III recent study, Telli et al. showed that preoperative combination of gemcitabine, carboplatin and iniparib is active in the treatment of early-stage triple negative and breast cancer 1/2 (BRCA1/2) mutation-associated BC [78]. A recent clinical trial reports results for veliparib, another PARP inhibitor, combined with carboplatin [79]. In TNBC, veliparib-carboplatin had an 88% predicted probability of success in a phase III trial. The estimated rate of pathological complete response in the TNBC population was 51% in the veliparib-carboplatin group added to standard therapy versus 26% in group treated with standard therapy (docetaxel, doxorubicin, and cyclophosphamide). The toxicity of veliparib-carboplatin was greater than in the group treated with standard therapy.

Other works have correlated TNBC subtypes, pCR status, and patient survival. As previously reported by Masuda et al., the basal-like 1 subtype of TNBC had the highest pCR rate (52%) as compared to basal-like 2 and luminal androgen receptor (LAR) subtypes (0 and 10%, respectively). However, TNBC mesenchymal subtype had the worst pCR and OS rates [80, 81]. Other studies such as the GeparTrio trial from the German Breast Group evaluated the androgen receptor (AR) expression in patients with primary BC treated with neoadjuvant docetaxel, doxorubicin, and cyclophosphamide. In this study, no significant difference between pCR rates of patients with AR+ TNBC tumors (29.2%) and those with AR− TNBC tumors (33.3%) were reported. However, AR+ patients had significantly better DFS and OS than those negative for AR [82]. However, in this study 22.5% of these patients relapsed after 5 years. Similarly, Yu et al. found that LAR subtype of TNBC, expressing high luminal genes (LAR and GATA3), had a relative favorable prognosis than tumors with cancer stem cell markers) [83]. Thus, targeting the AR in advanced TNBC offers a biologically promising strategy.

The partial responses of the above targeted molecules lead to the search for new therapeutic targets for TNBC, which is made possible by the BC genomes sequencing, which identified over 2414 somatic mutations, such as p53, PIK3CA and PTEN, which seem to be clonally dominant compared to other genes [84]. To our knowledge, no clinical trials targeting these genes in TNBC are ongoing.

## Cancer stem cells and therapeutic implications

Cancer stem cells (CSC), identified by several stem cell-like features including quiescence resulting in the escape of most cancer therapies, are characterized by their ability to self-renewal, to seed new tumors and should be capable of founding metastatic colonies after disseminating to foreign tissues [8, 85]. This is driven by the activation of the epithelia-to-mesenchymal transition (EMT) program, strongly involved in the dissemination of car-cinoma cells to distant tissues, through the deregulation of signaling pathways as Wnt/β-catenin, and conferred to CSC resistance to therapeutic agents [86, 87]. BC stem cells (BCSC), represent a small population of cells within the tumor mass exhibiting stem cell-like characteristics and have emerged as being responsible for tumor development, recurrence and MBC [88]. At the molecular level, these cells are characterized by the CD44+/CD24− phenotype and by the activation of signaling pathways, particularly those linked to the stem cell phenotype, such as nuclear factor-kappa beta (NF-κB), signal transducer and activator of transcription 3 (STAT3), Wnt/β catenin, Hedgehog, and NOTCH. In vitro and in patients, BCSC do not only exhibit intrinsic resistance to chemotherapy but they are also amplified following treatments [89, 90]. Clinical studies showed an increase of cells expressing CD44+/CD24− phenotype in primary tumors after chemotherapy [91]. Targeting the signaling pathways linked to stem cell phenotype should be of interest to antagonize BCSC. For instance, targeting glycogen synthase kinase 3 (GSK3)/β catenin signaling was sufficient to reduce the stemless features of BC cells [92]. Indeed, the LGK974, a porcupine inhibitor, which blocks Wnt palmitoylation [93], and PRI-724, CAMP responsive element binding protein(CREB)/catenin antagonist [94], have shown interesting results and are in phase I trial for breast cancer and other solid tumors. Some other inhibitors targeting NOTCH, NF-κB, STAT3 have shown their anti-tumor activity in vitro and in vivo models [88]. Clinical trials with inhibitors are only in their early stages.

Although there is an undeniable benefit of the therapies listed above, its absolute effect remains modest and depends on the molecular subtype of BC. There is therefore a need to identify more biomarkers that would make possible to select patients who benefit from these treatments or novel therapeutic agents. This approach comes up against the complexity of the tumor and its intra- or inter-tumor heterogeneity. The immune system appears among the components involved in this heterogeneity.

## Immune system and immunotherapy in BC

It is evidenced that host anti-tumor immunity can play a key role in the outcome of malignant cells including BC [95]. For a long time, breast cancer has been considered less immunogenic cancer as compared to melanoma and non-small lung cancer [96]. However, the presence of tumor infiltrating lymphocytes (TILs) in and around tumors and its correlation with improved pathological complete response and clinical survival have change our point of view [97].

Numerous studies confirm the presence of TILs in most primary BC, and this could influence prognosis and response to therapy. TILs rates seem to differ according to various subtypes [98, 99]. Indeed, TNBC and HER-positive breast cancer have generally higher TILs levels than ER+/HER− BC, suggesting that these subtypes are more immunogenic [100, 101]. The presence of immune cells within tumors may increase the likelihood of response to chemotherapy or immune checkpoint therapy. This has been confirmed in numerous types of cancers including BC [102]. A growing body of data in early-stage BC indicates that TILs-rich tumors exhibiting lower recurrence rates, improved response to neoadjuvant chemotherapy, and support immunotherapy with conventional therapy in future BC research. A meta-analysis including data from 2987 patients with early stage of BC indicated that TILs-rich tumors were associated with 30, 22, and 34% reduction of the risk of recurrence, distant recurrence, and death, respectively [103]. Moreover, TILs-rich tumors predicted superior overall survival benefit irrespective of the disease phenotype, particularly in early TNBC [104]. In a prospective-retrospective phase III trial that enrolled 1010 early-stage BC patients, Loi et al. showed that in TNBC (n = 134), increase in TILs was significantly associated with decreased distant recurrence. Moreover, the authors showed an association between higher levels of TILs and increased trastuzumab benefit in HER2+ disease [104]. These results are also confirmed by another study, this time based on the two phase III randomized adjuvant BC trials: ECOG 2197 and ECOG 1199, and two other adjuvant chemotherapy trials. Amongst 481 TNBC, 80% of tumors had at least 10% of stromal TILs (range 10–80%), but only 15% of cancers had at least 10% of intraepithelial TILs (range 10–50%), suggesting that stromal lymphocytic infiltration constitutes a robust prognostic factor in TNBC. Furthermore, the presence of TILs was associated with a better prognosis, with a reduction in the risk of relapse at a distance or deaths of 14% (p = 0.02) at each stage of 10% presence of TILs [105].

Dieci et al. also investigated whether lymphocyte infiltrates can predict benefit from adjuvant anthracyclines. They demonstrated that, in multivariate analysis, continuous TILs (intra-tumoral and stromal) were strong prognostic factors for OS, which was limited to TNBC and HER+ patients. Ten-year OS rates were 89 and 68% for TNBC high TIL and low TIL, respectively, and 78 and 57% for HER2+ high-TILs versus low-TILs, respectively. Moreover, TILs variability was not predictive for the efficacy of anthracyclines [106]. Similarly, Pruneri et al. reported, in the IBCSG phase III randomized clinical trial 22-00, that low-dose of oral ‘metronomic’ cyclophosphamide-methotrexate maintenance chemotherapy confers a greater but not statistically significant clinical benefit in patients with lymphocyte-predominant BC [107]. Another recent clinical trial showed that whether the high level of stromal TILs is beneficial to patients treated with standard chemotherapy (doxorubicin, cyclophosphamide followed by paclitaxel), the trastuzumab junction does not improve the anti-tumor response in HER2+ BC with high stromal TILs [108].

In neoadjuvant situations, the NeoALTTO trial (neoadjuvant Lapatinib and/or Trastuzumab treatment optimization) aimed at evaluating TILs in HER2+ BC (positive and negative RH). This study evaluated the addition of lapatinib to trastuzumab pre- and post-operative, combined with chemotherapy-based of paclitaxel and fluorouracil, epirubicin and cyclophosphamide [109]. Although, this study showed a benefit in terms of pCR, it was negative for OS and survival without event. Within this cohort, an evaluation of TILs out of a total of 395 tumors showed that the presence of more than 5% TILs was associated with a higher pCR. Similarly, for each increase in 1% of the TILs rate, event risk decreased by 3% [109].

An interesting study also evaluated TILs and immunologically relevant genes in the neoadjuvant GeparSixto trial in patients with HER2+ and TN [110]. In this study, the authors showed that TILs-rich tumors, defined by the presence of more than 60% of TILs at the level of stroma and tumor, accounted for 24.5% of the cohort and had a better response rate to neoadjuvant chemotherapy (carboplatin, anthracycline, and taxane combination), with pCR rates of 59.9% for these TILs-rich tumors versus 33.8% for tumors with less TILs. In the same way, the expression of immunity genes has been carried out. This analysis involved genes that have anti-tumor immunity (CXCL9, CCL5, CD8A, CD80, CXCL13, IGKC, and CD21) and genes having regulatory negative effects (IDO1, PD-1, PD-L1, CTLA-4, and FOXP3). Three groups of tumors could thus be identified according to the expression of these genes (low, intermediate and high), with rates of lymphocytic infiltration and pCR all the more large that these genes are overexpressed. The highest odds ratio was observed for PD-L1 and CCL5 [111].

Recently, Hendrickx and colleagues also analyzed the relationship between breast cancer genetic programs and antitumor immunity. They defined distinct immune phenotypes, e.g. the Th1 phenotype, marked by the highest immune gene expression, was associated with prolonged patients’ survival. In this immune favorable phenotype, mutations of *TP53* were enriched. Conversely, MAP3K1 and MAP2K4 mutations were associated with an immune-unfavorable phenotype [112].

Overall, most results are consistent with the notion of improved benefits to trastuzumab in those with higher TILs in patients with HER2+ and TNBC.

The evaluation of TILs within tumors could in future be integrated as a new parameter in therapeutic decision-making. However, it is necessary to standardize this evaluation by defining the tumor surface to be analyzed (in order to be representative), or to choose the threshold of the % TILs.

But in tumors where TILs are poorly present or absent, strategies should be devised to increase immune effectors within the tumor. Some are currently under study.

## Immune checkpoint blockade

Immune checkpoint blockade has shown its usefulness by the impressive survival benefits seen in phase III trials with anti-cytotoxic T-lymphocyte-associated protein 4 (CTLA4) and anti-programmed cell death protein-1 (PD-1) therapy in [113, 114], non-small cell lung cancer [115, 116], and renal cell carcinoma [117]. In contrast, there are less reported outcomes of checkpoint blockade efficacy in BC [118]. Nevertheless, there are some arguments for adapting the immunotherapy checkpoint to BC, such as results that show that PD-1 expression in tumor stroma in BC has been shown to be associated with an aggressive phenotype in terms of prognosis and is more common in TNBC [119]. Among the few clinical trials developed, we can cite a first trial that used anti-CTLA4 immunotherapy in patients with advanced BC in combination with the aromatase inhibitor exemestane [120]. In this study, the best OS was stable disease for >12 weeks in 11 patients (42%). There is an association in most patients between treatment efficacy and increase in peripheral CD4+ and CD8+ T cells expressing inducible T-cell costimulator (ICOS), and in the ratio of ICOS+ T cells to forkhead box P3 (FoxP3)+ regulatory T cells. Two other Phase I clinical trials have shown promising results in patients with TNBC, the first tested pembrolizumab (MK-3475), an anti-PD-1 antibody, in 27 affected patients of TN MBC expressing PD-L1, and heavily pretreated, the response rate was of 18%, including one complete response and four partial responses, three patients were treated for more than 12 months [121]. A second Phase I trial was presented to the American Association for Cancer Research’s 2015 Annual Meeting, and tested an anti-PD-L1 antibody, MPDL3280A (atezolizumab). In 54 patients with metastatic TNBC, of which 69% were PD-L1 positive, tolerance was good, with 36 patients (66%), reporting side effects of all grades with fatigue, fever and nausea, and six (11%) of serious side effects (type of insufficiency adrenal, nausea, anemia and grade neutropenia 3–4). The objective response rate was 24%, two complete responses and three partial responses. Similarly as in the previous trial, prolonged responses were observed in six patients (29%) with survival without progression (PFS, progression free survival) to more than 24 weeks [122].

## Conclusions

It is clear that the characterization of new mutation and driver genes have allowed the development of new targeted therapeutic strategies (targeting these genes) in combination with standard chemotherapies used in the treatment of breast cancer. Despite advances in innovative clinical trial designs, challenges persist, such as the intratumoral and intertumoral heterogeneity. To address such challenges, efforts are still need to find new targets therapeutic detected particularly at low frequency, and the development in parallel of biomarkers that will result in the improvement of patient outcomes in BC medicine.

## Abbreviations

WHO: World Health Organization
ER: estrogen receptor
PgR: progesterone receptor
AR: androgen receptor
HER2: the oncogenic human epidermal growth factor receptor 2
PTEN: phosphatase and tensin homolog
PIK3CA: phosphoinositide-3-kinase catalytic alpha polypeptide
Ki67: Ki67 antigen
BC: breast cancer
BCS: breast-conserving surgery
BCT: breast-conserving therapy
pCR: pathologic complete response
MBC: metastatic breast cancer
AI: aromatase inhibitor
Pi3K: phosphatidylinositol-3-kinase
Akt: protein kinase B
mTOR: mammalian target of rapamycin
ESR1: estrogen receptor gene 1
mTORC2: mammalian target of rapamycin complex 2
DNA: deoxyribonucleic acid
CCND1: gene encodes the cyclin D1 protein
RB: retinoblastoma protein
CDKN2A: cyclin-dependent kinase inhibitor 2A
PFS: progression-free survival
CDK4/6: cyclin-dependent kinase 4/6
INPP4-B: inositol polyphosphate 3-kinase-phosphatase, type II
TNBC: triple-negative breast cancer
EGFR: epidermal growth factor receptor
IGF-1R: insulin-like growth factor 1
MET: met prot-oncogene
ERBB receptors: include EGFR family receptors
PARP: poly(adenosine diphosphate-ribose) polymerase
BCSC: breast cancer stem cells
EMT: epithelia-to-mesenchymal transition
NF-κB: nuclear factor κB
STAT3: signal transducer and activator of transcription 3
Wnt: wingless
TILs: tumor infiltrating lymphocytes
OS: overall survival
DFS: disease-free survival
PD-1: programmed cell death protein-1
PD-L1: programmed-death ligand 1
CTLA4: cytotoxic T-lymphocyte-associated protein 4
ICOS: inducible T cell costimulator
GM-CSF: granulocyte macrophage colony stimulating factor
HLA: human leucocyte antigen
